# Clinical, Histopathological, and Management Challenges of Multiple Familial Trichoepithelioma: A Case Report of a Patient Presenting with Multiple Facial Papules

**DOI:** 10.1155/2020/5648647

**Published:** 2020-09-07

**Authors:** D. W. V. N. Dissanayaka, D. K. B. Dassanayaka, P. R. Jayasooriya

**Affiliations:** ^1^Department of Oral Pathology, Faculty of Dental Sciences, University of Peradeniya, Sri Lanka; ^2^Oral and Maxillofacial Surgery Unit, Provincial General Hospital Badulla, Sri Lanka

## Abstract

Trichoepitheliomas (TE) are benign skin tumours of the pilosebaceous apocrine unit with follicular differentiation. Multiple familial trichoepithelioma (MFT) is a considerably rare condition inherited in an autosomal dominant pattern. We present a case of a 15-year-old male who presented with multiple papulo-nodular lesions in the central face and a family history of a similar type of lesions from his mother. Significance of consideration of various clinical differential diagnoses with serious pathological outcomes, strategies followed in the diagnosis including histopathological evaluation aided by immunohistochemical investigations, and subsequent challenges that may be faced in the management of MFT in light of the presentation with multiple facial papules are documented in this case report.

## 1. Introduction

Trichoepithelial tumours are a group of benign, follicular, epithelial-stromal tumours, classified as classic trichoepitheliomas, trichoblastomas, trichoadenomas, and desmoplastic trichoepitheliomas [1]. Classic TE presents as familial multiple form or solitary form. MFT is inherited in an autosomal dominant fashion with lessened expressivity and penetrance in males [2]. The familial multiple form has been first described by Brooke under the title of *Epithelioma Adenoides Cysticum* in 1892 [3] and by Fordyce as “Multiple Benign Cystic Epithelioma” [4]. The exact prevalence of TE remains unknown, with the presentation of MFT being considerably rare [3]. Mutations in cylindromatosis tumour suppressor gene (CYLD), which maps to chromosome 16q12-q13, is accounted to lead to TE [5].

In MFT, lesions appear predominantly on the face, neck, scalp, trunk, and upper extremities. Eyebrows, cheeks, and eyelids are often involved [1–3]. Onset is usually seen in the childhood as asymptomatic, translucent, firm, papulo-nodular lesions predominantly affecting naso-labial folds and the central part of the face [6, 7]. Lesions are often roughly symmetrical in shape and distribution and increase in size and number with age, producing a significant cosmetic disfigurement. They may coalesce to form large lesions, become so thickly grouped together as to form raised lumpy patches of a disfiguring leonine facies [8], or may slowly enlarge to a maximum diameter of 0.5 cm and rarely ulcerate at late stages [1, 2, 8, 9].

This case report describes a patient who presented with multiple facial papules and with a family history of the same condition from a first-degree relative, diagnosed as MFT. The clinical differential diagnoses and histological diagnostic dilemma associated with the presentation as asymptomatic, multiple facial papules and therapeutic challenges will be discussed in this case report.

## 2. Case Report

**Figure 1:**
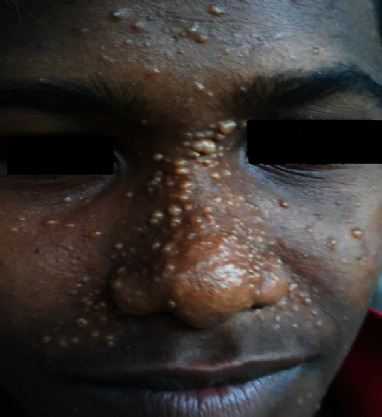
A 15-year-old patient presenting with multiple facial papules.

A 15-year-old male patient presented with multiple asymptomatic nodules in relation to the nose, nasolabial folds, and temporal and frontal areas of the face (Figure 1). The patient had noticed the first lesion over his left nasolabial fold at the age of 5 years.

**Figure 2:**
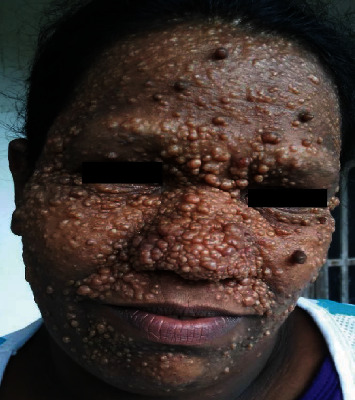
Patient's mother with multiple facial papules relatively larger in size and extensively distributed over the entire face.

**Figure 3:**
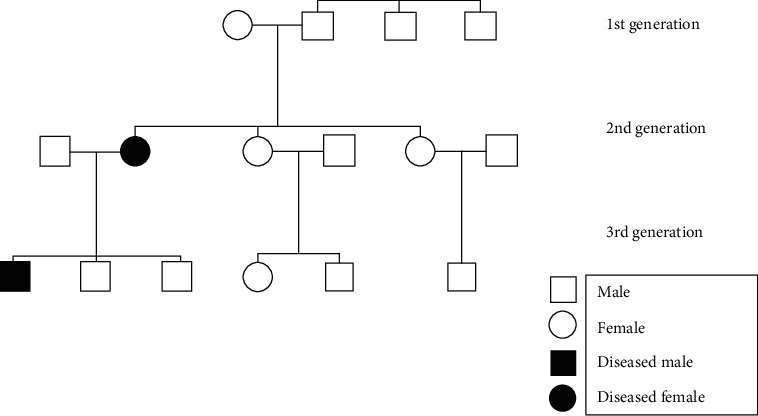
Pedigree of the patient showing hereditary pattern of trichoepitheliomas.

Thereafter, lesions had gradually increased in size and number with the increase being more evident for the last two years. The patient's 38-year-old mother had been reported with multiple, similar lesions symmetrically and extensively distributed on the entire face (Figure 2). Her lesions had started appearing around puberty on the central parts of her face and gradually increased in size and number and were distributed over her entire face (Figure 3).

**Figure 4:**
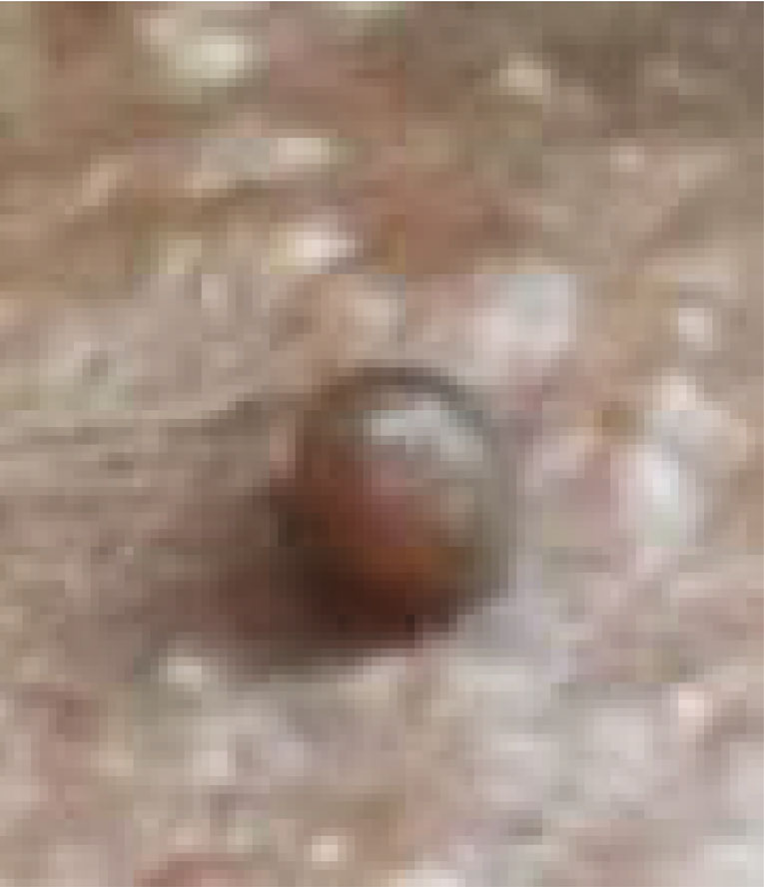
A single well-circumscribed and translucent papule.

On examination, lesions were multiple, firm in consistency, and well circumscribed, with translucent papules and nodules ranging in size from 0.5 to 2 cm (Figure 4). The center of some lesions was slightly depressed and umbilicated. The lesions were distributed predominantly over the central face in a symmetrical pattern.

The patient was otherwise healthy looking without any lymphadenopathy, and papules were not revealed in other sites of the body such as the scalp, neck, and trunk. A number of clinical differential diagnoses such as neurofibromatosis-1, Cowden's syndrome, tuberous sclerosis, multiple endocrine neoplasia type IIB, multiple familial trichoepitheliomas (MFT), and Brooke-Spiegler syndrome (BSS) were derived (Table 1), and subsequently, biopsies were performed.

**Figure 5:**
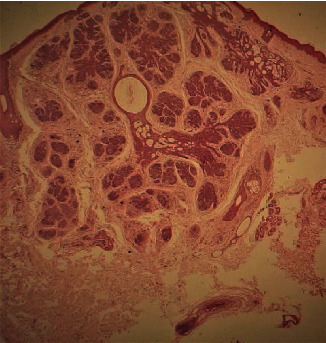
Pictomicrograph of the symmetrical adnexal tumour composed of lobules of basaloid cells and keratocytes (H&E, low power, ∗10).

**Figure 6:**
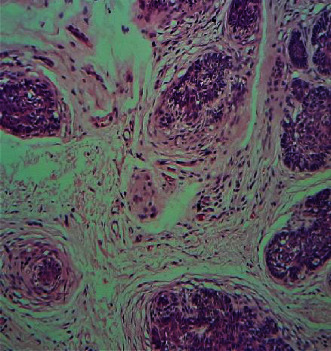
Prominent perilobular connective tissue sheath (H&E, high power, ∗40).

**Figure 7:**
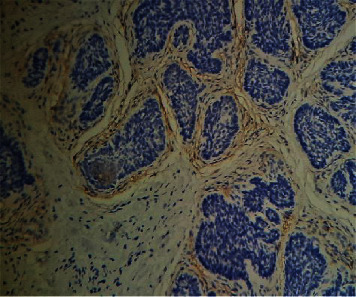
Peripheral stromal lining of the tumour positively stained with CD10 (H&E, high power, ∗40).

Histopathological analysis of the lesion revealed a symmetrical adnexal tumour predominantly composed of lobules of basaloid cells (Figure 5) with focal horn cysts and keratinization. In a few foci, prominent perilobular connective tissue sheaths were noted (Figure 6). The aforementioned histopathological features were consistent with those of trichoepithelioma (TE), and further immunohistochemical analysis with CD10 revealed positivity only in the stromal lining (Figure 7). The positive family history, presence of multiple lesions, and histopathological features along with immunohistochemical analysis were consistent with those of TE, and multiple familial trichoepithelioma syndrome was suggested to be the most likely diagnosis. However, confirmatory genetic analysis was not possible due to unavailability of facilities and financial constraints.

**Figure 8:**
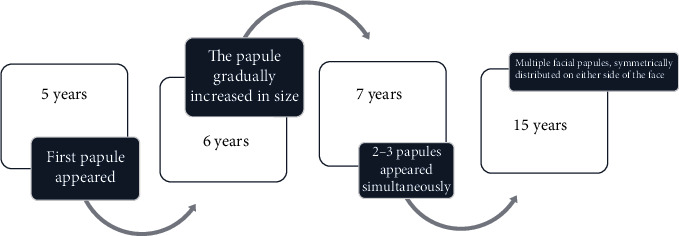
Timeline of the disease process.

Upon confirmation of the diagnosis as MFT, the treatment plan was to excise relatively large papules surgically, and relatively smaller lesions were to be managed with 5% Imiquimod topical therapy for 32 weeks (Figure 8). Unfortunately, the patient did not attend the clinic following the diagnosis, most probably due to travel restrictions enforced due to the COVID-19 pandemic.

## 3. Discussion

In the present case, the patient had a positive family history of similar presentation with the patient's mother. The patient is the oldest in the family of three boys, and no other family member was reported to be affected with any adnexal tumours. Autosomal dominant transmission was most likely, with possible sporadic mutation occurring in the patient's mother (Figure 4). Furthermore, less expressivity and penetrance of the CYLD gene mutation was accountable in this case, since the son presented with lesions which are comparatively smaller in size and confined to only the central part of the face.

MFT appears around puberty or late childhood as multiple, firm, rounded, translucent, shiny, well-demarcated papulo-nodular lesions. The lesions are symmetrical in distribution, more apparent in the space between the eyebrows, the root of the nose, the nostrils and naso-labial folds, the upper lip, and, to a lesser extent, the chin, similar to our patient. There are no significant systemic associations in patients presenting with MFT with the case presented in this case report.

Several clinical differential diagnoses could be taken into consideration, when a patient presents with multiple facial papules as indicated in Table 1.

Histopathological evaluation was essential to derive a definitive diagnosis for appropriate management because many disease entities, as listed in Table 1, may produce multiple facial papules. The other conditions that are considered in clinical differential diagnoses such as Cowden's syndrome, Muir-Torre syndrome, tubular sclerosis, and Bazex-Dupre-Christol syndrome have serious underlying pathological consequences such as an increased risk for developing malignancies. Thus, accurate diagnosis as early as possible would lead to proper management and improved survival rates. Dental surgeons should consider the possibility of the aforementioned diseases with underlying serious pathological consequences in patients who present with multiple facial papules.

Brooke-Spiegler syndrome (BSS) (Online Mendelian Inheritance in Man (MIM# 605041)) has an autosomal dominantly inherited predisposition towards skin appendage tumours including cylindromas, TEs, and/or spiradenomas. Familial cylindromatosis (FC) and MFT are accounted to represent two ends within the spectrum of Brooke-Spiegler syndrome [10], but the patient presented in this case report did not present with spiradenomas which are purple to bluish nodules that are paroxysmally painful when symptomatic, usually located on the trunk and extremities, and cylindromas which are slow-growing tumours that typically occur on the scalp. Hence BSS was excluded.

Histologically, a diagnostic dilemma accounts in the distinction between TE and basal cell carcinoma (BCC). Histologically, TE exhibits a more organoid growth pattern than BCC and consists of nests of basaloid cells of similar size with follicular differentiation embedded within tumour stroma that dissects through the collagen into the upper reticular dermis. The fibrous matrix of TE is apparently different from the fibromyxoid stroma of BCC. Contrasting histologic features of TE include presence of papillary mesenchymal bodies, which is occasionally encountered in keratotic BCC and hair bulb formation. The presence of clefting between the epithelial and stromal components of the tumour or broad connections to the epidermis, stromal retraction, peripheral palisading of keratinocytes, single-cell necrosis, and brisk mitotic rate are associated with BCCs.

Rarely, BCC may develop in TE [11].The histopathological differentiation entirely based on haematoxylin and eosin staining is challenging since both tumours consist of basaloid cells with follicular differentiation. Hence, the pattern of expression of Bcl-2 and CD34 can be used to differentiate TE and BCC [12–14]. Bcl-2 is expressed in all cells in most BCCs but only weakly in the basal cell layer of some aggressive variants of TEs [14]. CD34 is found in the peritumoural fibroblasts around TE but not in the tumour stroma of BCC. Recently, CD10 is also reported to be used since basaloid cells are stained in BCC and stromal staining is present in TE which resolves histopathological challenge in the differentiation of the two entities (Figure 4).

MFTs on the face present a major cosmetic concern, and the management of TEs is challenging especially in patients with multiple facial lesions like the presented case. Various treatment modalities have been efficacious in treating TEs such as surgery, electrodessication, dermoabrasion, CO_2_ laser, Erbium-YAG laser, and Imiquimod [15]. Traditional therapy with surgery has been proven to be effective with solitary lesions but causes high rates of scarring and poor cosmetic outcome in multiple lesions. Laser irradiation has been accounted to have a good outcome as in the treatment of other cutaneous [16] and mucosal [17] lesions as they offer less pain and fast recovery [15]. However, management of multiple lesions is a notoriously difficult endeavor and all treatment methods carry a significant risk of side effects such as erythema, oedema, hyperpigmentation, and most importantly scarring [15]. Recurrences are also very common.

Imiquimod is an immunomodulating agent approved for the treatment of HPV infections, actinic keratoses, and basal cell carcinomas [15]. Several studies have reported the efficacy of Imiquimod in partial size reduction of lesions [18]. Topical therapy with Imiquimod is a relatively low-cost treatment with tolerable side effects and a high cure rate, considerably in patients with multiple lesions. However, the combination of surgical excision of relatively large lesions and topical treatment of relatively small lesions with Imiquimod was planned in this patient to enhance the treatment outcome. (Table 2).

## 4. Conclusion

Presentation with multiple facial papules poses a significant diagnostic challenge. Diagnosis is based on family history, clinical examination, and histopathological evaluation which is essential to exclude serious underlying pathological conditions. Long-term vigilance and follow-up for BCC development is warranted in MFT. It is essential to achieve a definitive diagnosis to facilitate appropriate management in a patient presenting with multiple facial papules since this presentation may be a part of an underlying syndrome with serious pathological consequences.

Dental surgeons, as health professionals, should consider the possibility of syndromes in patients who initially present with asymptomatic multiple facial papules and should contribute to early diagnosis of associated life-threatening conditions, facilitating effective treatment outcomes, increased survival rates, and quality of life.

## Figures and Tables

**Table 1:** Differential diagnoses for multiple facial papules.

Differential diagnoses for multiple facial papules	Clinical features	Significance
Brooke-Spiegler syndrome [19, 20]	Multiple trichoepitheliomas, cylindromas, and spiradenomas	Parotid basal cell adenomas, adenocarcinomas

Multiple familial trichoepitheliomas [1, 2, 21]	Multiple lesions with autosomal dominant inheritance	Malignant transformation to basal cell carcinomas

Familial cylindromatosis [3, 9]	Multiple skin tumours	Vision difficulties

Cowden's syndrome [22]	Multiple trichilemmomas, fibroepithelial polyps, and macrocephaly	Increased risk of developing malignancies in the breast, thyroid, and endometrium

Birt-Hogg-Dube [23]	Fibrofolliculomas, acrochordons, angiofibromas, oral papules, cutaneous collagenomas, and epidermal cysts	Pulmonary cysts/history of pneumothorax, and various types of renal tumours

Rombo's syndrome [24]	Multiple trichoepitheliomas, milia, and vermiculate atrophy	Basal cell carcinoma, peripheral vasodilation, and cyanosis

Bazex-Dupre-Christol syndrome [25, 26]	Congenital hypotrichosis, follicular atrophoderma, milia, basal cell carcinomas, and hypohidrosis	Presence of a primary malignant neoplasm of the upper aerodigestive tract or metastatic cancer to the lymph nodes of the neck

Muir–Torre syndrome [26, 27]	Sebaceous adenomas, keratoacanthomas	Colonic polyposis, occult laryngeal carcinomas, and gastric carcinomas

Tuberous sclerosis [26]	Multiple cutaneous angiofibromas, ash leaf spots, cardiac rhabdomyoma, and cortical tubers	Patients suffering from various symptoms, organs affected by tumours, and mentally retarded

Neurofibromatosis-1 [26]	Multiple neurofibromas, plexiform neurofibromas, café-au-lait patches, axillary freckling, and Lisch nodules	Neurofibromas may undergo malignant transformation

Gardener syndrome [26]	Multiple osteomas, epidermoid cysts, supernumerary teeth, and odontomes	High-malignant transformation rate for gastrointestinal polyps

MENIIB [26]	Multiple mucosal neuromas, phaeochromocytomas, and thyroid malignancies	Oral/perioral mucosal neuromas develop prior to malignancies and would help in early diagnosis

**Table 2:** Summarization of followed therapeutic regimen and duration.

32 weeks	Topical treatment with 5% Imiquimod
36 weeks	Surgical excision of relatively larger remaining lesions
42 weeks	Healing of surgical sites with scarring
After 42 weeks	Second cycle of treatment with 5% Imiquimod
